# B-cell diversity decreases in old age and is correlated with poor health status

**DOI:** 10.1111/j.1474-9726.2008.00443.x

**Published:** 2009-02

**Authors:** Kate L Gibson, Yu-Chang Wu, Yvonne Barnett, Orla Duggan, Robert Vaughan, Elli Kondeatis, Bengt-Olof Nilsson, Anders Wikby, David Kipling, Deborah K Dunn-Walters

**Affiliations:** 1 Department of Immunobiology, King's College London Medical SchoolLondon, UK; 2 School of Science and Technology, Nottingham Trent UniversityNottingham, UK; 3 Clinical Transplantation Laboratory, Guys HospitalLondon, UK; 4 Department of Infectious Diseases, Ryhov HospitalJonkoping, Sweden; 5 Division of Infectious Diseases, Department of Molecular and Clinical Medicine, Faculty of Health Sciences, Linköping UniversityLinköping, Sweden; 6 Department of Natural Science and Biomedicine, School of Health Sciences, Jönköping UniversitySweden; 7 Department of Pathology, School of Medicine, Cardiff UniversityCardiff, UK

**Keywords:** aging, B cells, diversity, elderly, immune frailty

## Abstract

Older people suffer from a decline in immune system, which affects their ability to respond to infections and to raise efficient responses to vaccines. Effective and specific antibodies in responses from older individuals are decreased in favour of non-specific antibody production. We investigated the B-cell repertoire in DNA samples from peripheral blood of individuals aged 86–94 years, and a control group aged 19–54 years, using spectratype analysis of the IGHV complementarity determining region (CDR)3. We found that a proportion of older individuals had a dramatic collapse in their B-cell repertoire diversity. Sequencing of polymerase chain reaction products from a selection of samples indicated that this loss of diversity was characterized by clonal expansions of B cells *in vivo.* Statistical analysis of the spectratypes enabled objective comparisons and showed that loss of diversity correlated very strongly with the general health status of the individuals; a distorted spectratype can be used to predict frailty. Correlations with survival and vitamin B12 status were also seen. We conclude that B-cell diversity can decrease dramatically with age and may have important implications for the immune health of older people. B-cell immune frailty is also a marker of general frailty.

## Introduction

Older people suffer from a decline in the immune system that affects their ability to respond to infections and to raise effective responses to vaccines (Weinberger *et al.*, 2008). This is particularly apparent from the high mortality rates due to pneumonia in the elderly (Office for National Statistics, 2005), and their susceptibility to hospital-acquired infections, such as *Clostridium difficile* and methicillin-resistant *Staphylococcus aureus* (Health Protection Agency, 2008). Many aspects of the immune system are altered in immunosenescence. The T-cell repertoire is decreased, T cells lose responsiveness (Effros *et al.*, 2003; Koch *et al.*, 2006), macrophages have a decreased antigen-presenting capacity and altered cytokine secretion (Dace & Apte, 2008), follicular dendritic cells cannot present antigen as efficiently (Aydar *et al.*, 2004), and neutrophils lose phagocytic ability (Lord *et al.*, 2001). The humoral immune system is crucial to the effective response against bacterial infections such as pneumonia, but its role in immune frailty has not been well studied. It is known that the quality of the antibody response changes with age, with a lower level of specific antibodies and an increased level of non-specific antibodies being generated in response to vaccination (Howard *et al.*, 2006). The reasons for this failure, and the increased susceptibility to bacterial infections, remain to be fully elucidated, but it seems likely that changes in the humoral immune system would play a key role in the increased immune frailty of the elderly.

High-affinity specific antibodies are generated during affinity maturation, a process that takes place in the germinal centre of secondary lymphoid tissue and is characterized by somatic hypermutation of Ig genes and subsequent selection of the genes encoding the best antibodies (MacLennan, 1994). There have been reports of an increased level of mutations in Ig genes in older people (Dunn-Walters *et al.*, 1997; Chong *et al.*, 2003). However, we have shown that the somatic hypermutation process occurs at the same rate in young and old humans, and so this difference is more likely a consequence of accumulation rather than altered rate (Banerjee *et al.*, 2002). We have also found that the imprint of hypermutation, in terms of the types of mutations and the hotspots in which they occur, is the same in samples from both young and elderly individuals, suggesting that the mechanism of hypermutation does not change with age (data not shown). Finally, data from our study (Banerjee *et al.*, 2000) and that of others (Lazuardi *et al.*, 2005) provide no evidence for age-related changes in the size and number of germinal centres in humans, although the situation may be somewhat different in mice (Zheng *et al.*, 1997).

Although the most well-known function of B cells is as antibody producers, they have intrinsic properties that also make a vital contribution to the immune system. They are highly effective as antigen-presenting cells, and have been shown to be essential for the development of T-cell memory (Crawford *et al.*, 2006). There is also now substantial evidence to support their role as immune regulators, since they are capable of secreting IL10. IL10-secreting B cells could serve to prevent inappropriate stimulation of the immune system, such as that leading to autoimmune disease, and could also serve to limit the aggressiveness of bona fide immune responses (Fillatreau *et al.*, 2008). It thereby follows that a loss of diversity in the B-cell repertoire would be predicted to have dramatic and serious consequences for the integrity of the humoral immune system. To date there has been a paucity of evidence regarding changes in the B-cell repertoire with age. Some contradictory studies have concentrated on determining whether there is a change in the relative proportions of the different IGHV gene families with age (Van Dijk-Hard *et al.*, 1997; Wang & Stollar, 1999), but since there are only six different IGHV families, and an estimated 10^8^ B cells per person, this method of assessing diversity is less than ideal. Studies focussing on sequencing the area of the hypervariable complementarity determining three (CDR3) region of the IGH gene, an area highly important for antigen binding, have been limited by the logistics of large-scale sequencing and so have been restricted to small numbers of individuals and only one subgroup of Ig genes (Xue *et al.*, 1997; Kolar *et al.*, 2006). Because these and other previous studies were restricted in their ability to measure diversity, we therefore set out to undertake a comprehensive analysis of B-cell diversity in the elderly, using as our technique the CDR3 spectratyping of all IGH genes. The CDR3 region of the Ig heavy chain gene comprises the area where three different Ig genes (IGHV, IGHD, IGHJ) join together to make the whole. As such it is highly diverse, both in sequence and in the length of the sequence. Amplifying the CDR3 region of the Ig gene using labelled primers and running the subsequent products on a high-resolution sequencing gel produces a characteristic spectratype, representing the distribution of different CDR3 sizes in the sample population. For our study population, we used peripheral blood mononuclear cell samples taken from older people in the Swedish NONA longitudinal study on immunosenescence. This study, and its predecessor (OCTO study), followed the health and T-cell immune characteristics of a number of old volunteers and resulted in the discovery of a T-cell ‘Immune Risk Phenotype’ (IRP) that predicted mortality in the very old. IRP is characterized by an expansion of CD8^+^ T cells, and a resulting inverted CD4^+^/CD8^+^ ratio (Wikby *et al.*, 1998, 2002). Later studies on the same patient group showed that inflammatory markers, such as IL-6, CRP and albumin, are also significant predictors of mortality in very old humans (Wikby *et al.*, 2006). Using an objective measurement of our B-cell spectratypes as a measure of B-cell diversity, together with additional direct sequence data, our data revealed a striking collapse in B-cell diversity in a subset of older individuals in this study that is not seen in our control samples from young, healthy individuals. Furthermore, this collapse in B-cell diversity is a biomarker that is strongly predictive of poor health status (frail vs. healthy) in the NONA cohort, as well as the likelihood of death within the next 4-year interval. Overall these data reveal a hitherto unexpected collapse in the humoral immune system with age in some elderly individuals, with significant implications for their ability to defend against pathogens and respond to vaccination.

## Results

### B-cell diversity can dramatically collapse in some older individuals

**Fig. 1:**
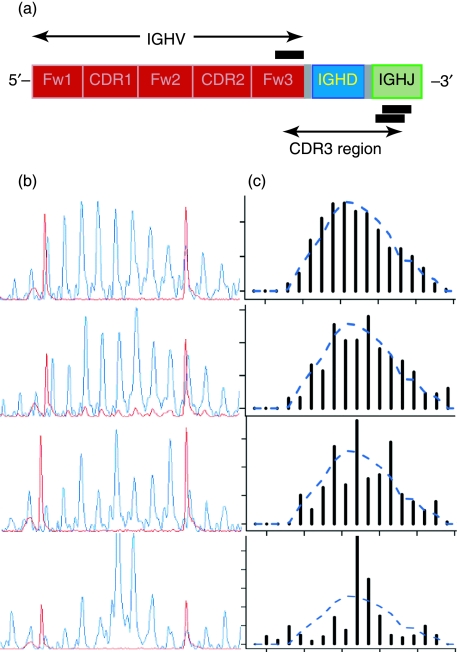
Generation of B-cell spectratypes from peripheral blood mononuclear cells. (a) Seminested PCR is performed over the CDR3 region (IGHV-IGHD-IGHJ joining region of the immunoglobulin heavy chain gene), the positions of the primers are indicated by the black bars. The 3′ nested primer is labelled with 6-Fam. The PCR products are electrophoresed on an ABI377 slab gel sequencing system to generate spectratypes (b). The heights of the individual peaks were determined and used to generate graphs where each fragment size is represented as a percentage of the whole (c). The blue dotted line overlaid on the plots represents the median distribution of the young control data set (aged 19–55 years).

We investigated the diversity of the peripheral blood B-cell population in older people using spectratype size analysis of the IGH CDR3 region (Fig. 1a). A polyclonal B-cell population from a healthy young individual typically yields a spectratype of CDR3 lengths in the range 57–117 bp that follows an approximately normal distribution (top panel in Fig. 1b,c). Although many individuals in the Swedish longitudinal NONA cohort also show a similar normally distributed spectratype, we observed several samples where the spectratype was strikingly and reproducibly non-normal (third and fourth panels in Fig. 1b,c), often due to the presence of one or more unusually abundant fragment lengths.

**Fig. 2:**
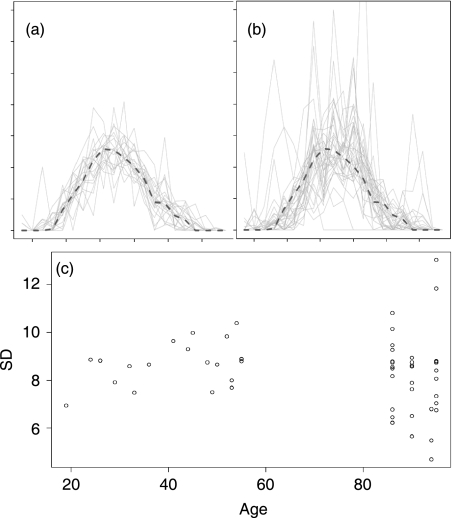
Age effects on B-cell spectratypes. The line plots for B-cell spectratypes from peripheral blood mononuclear cells of 24 young (a, aged 19–55 years) and 32 old (b, aged 86–94 years) donors are overlaid to show the increased deviation from a normal distribution in some old samples. The dotted line overlaid on the plots represents the median distribution of the young control data set (aged 19–55 years). The SD values for each sample individually are shown, plotted against the donor's age (c).

To compare multiple spectratypes, the fragment size and peak height of the initial ABI 377 spectratype data were quantified, which enable the data to be summarized in graphical format (Fig. 1c). Using this summarization technique we compared data from blood samples from a younger control group (28 people, 19–55 years old) with samples from 45 participants of the NONA study (aged 86, 90 and 94 years). The shape of the spectratypes varied much more in the older population than in the younger control group, as can be clearly seen when all the data are overlayed (Fig. 2a,b).

The presence of peaks of altered height in such spectratypes distorts the fragment distribution away from a normal (Gaussian) distribution, such as that seen in the young samples. A number of parameters could be used to explore these distributional changes, including skew and kurtosis, or the correlation to a normal distribution. However, a simple metric that is sensitive to these distortions is the standard deviation (SD) of the distribution. This decreases in instances (such as the bottom panels in Fig. 1b,c) where there is increased peak height in fragments close to the centre point of the distribution, and increases in those cases when the peak height increases are in fragment lengths that are towards the edge of the range. When plotted vs. age, it is striking that whereas the mean of the SDs of both the young and NONA samples are approximately equal, the latter show a significantly wider spread of SD values (Fig. 2c).

**Fig. 3:**
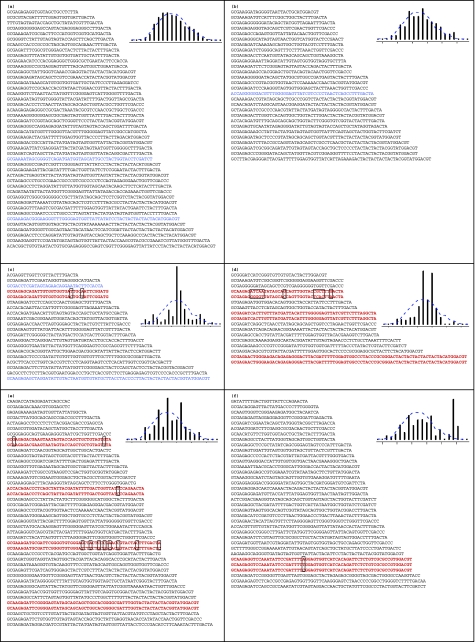
Clonal expansions of B cells in samples with distorted spectratypes, but not in samples with normal spectratypes. The CDR3 regions, covering the IGHV-IGHD-IGHJ joining regions of the immunoglobulin heavy chain gene, were cloned and sequenced from six samples. Sequences highlighted in red are examples of clonal expansions, with the same sequences found in different cloning reactions, or with evidence of intraclonal heterogeneity such as is created by somatic hypermutation during affinity maturation. The spectratype for each sample is shown next to the sequence grouping. Samples A and B are donors aged 95 and 86, respectively, and who have normally-distributed spectratypes. Samples C, D, E and F were aged 90, 86, 86 and 95 years, respectively, and all had distorted spectratypes.

The simplest hypothesis is that the altered peak heights in a distorted spectratype represent reduced B-cell diversity, together with possible oligoclonal expansions in the B-cell repertoire. To test this we chose six samples from older individuals for further investigation, two of which had a spectratype of approximately normal appearance and four that deviated markedly from a normal distribution. Polymerase chain reaction (PCR) products from these samples were sequenced in order to more closely examine the diversity of the population (Fig. 3). In the samples that showed distorted (non-normal) spectratypes, we observed multiple independent identical, or near-identical, sequences (Fig. 3). In addition there are some sequences that have the same V-D-J rearrangement but show individual nucleotide differences; these differences are due to *in vivo* somatic hypermutation events since the observed level of hypermutation is far higher than the experimentally determined PCR error rate for these methods (Dunn-Walters *et al.*, 1995). These duplicates thus represent *in vivo* clonal expansions of an individual B-cell family, and overall these data provide strong evidence for oligoclonality (and thus a collapse in B-cell diversity) in those samples with spectratypes of non-normal appearance.

### The extent of loss in B-cell diversity correlates with health status in the elderly

**Fig. 4:**
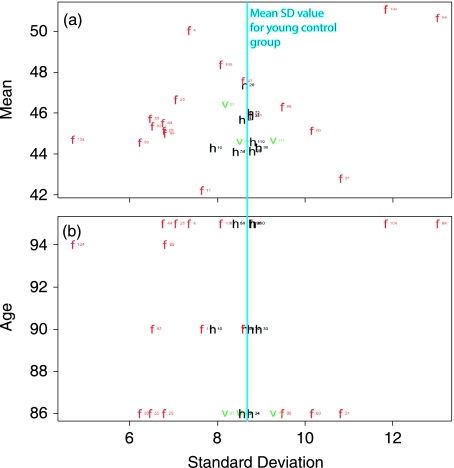
Visualization of the relationship between health status and spectratype SD. The SD of each spectratype was calculated as the measure of its distortion and then plotted vs. mean fragment length for ease of visualization (a). Each point is labelled according to the previously determined (Wikby *et al.*, 2002) health data for the donors. vh, very healthy; h, healthy; f, frail. The majority of individuals that are ‘h’ or ‘vh’ have spectratype SDs that are close to mean SD value determined for the younger control group. In contrast, those samples with unusually low or high SDs are entirely those people classified as frail. This observation was independent of the age of the donor (b).

The results presented above indicated that many of the NONA individuals had distorted spectratypes whose SD was smaller, or larger, than that generally seen for the young population (Fig. 2c). To further explore our data we superimposed the health status of each individual upon a plot of the SDs of the NONA samples (Fig. 4). Initial visualization revealed that those spectratypes whose SD values were atypically low or high when compared to the mean SD for the control group were also the samples from volunteers that had been classified as frail at the time of sampling (Wikby *et al.*, 2002), whereas those classified as healthy, or very healthy, generally had spectratype SD values closer to the mean SD of the control group spectratypes (Fig. 4a). This effect was seen for each of the three older age groups individually (Fig. 4b).

**Fig. 5:**
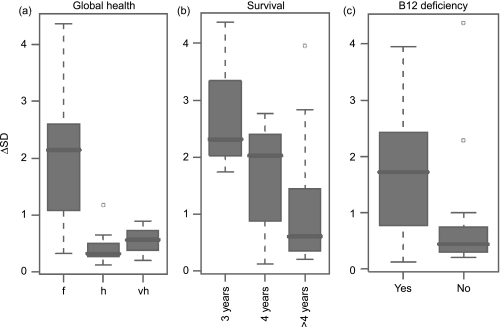
Correlations with health and survival. The ΔSD value for each sample was determined as being the difference between the sample value and the mean control value. Donors had previously been classified as frail (f), healthy (h) or very healthy (vh) (Wikby *et al.*, 2002). The difference between the healthy/very healthy and the frail was significant (*p* = 0.00006 by MWU), as was the difference between people who survived longer than 4 years and those that passed away within 3 years (b, *p* = 0.035 by MWU). We also found a significant difference in ΔSD between patients with and without vitamin B12 deficiency (c, *p* = 0.026 by MWU).

To provide a single metric that is linearly related to this degree of deviation from the expected SD, we therefore calculated the difference (in absolute terms) between the sample SD and the mean SD of the control group spectratypes. This metric, referred to as ΔSD, provides a singal value reflecting the degree of distortion of a spectratype and thus diversity, with high values associated with restricted diversity. Fig. 5(a) provides a different summarization of Fig. 4, and demonstrates that an elevated ΔSD value is associated with frailty. The difference between the frail group and the others is highly significant [*p* < 0.0001 by Mann–Whitney *U*-test (MWU)]. The ΔSD value is predictive of fraility, with ΔSD of greater than 1 predicting frailty with a sensitivity of 0.78 and a specificity of 0.92 [as determined by receiver operating characteristic (ROC) curve analysis].

Finally, since the blood samples had been taken several years previously, it was possible to utilize follow-up data on survival of the donors. This revealed that there was also a significant difference in ΔSD between those that survived longer than 4 years and those that died within 3 years after donating the sample for analysis (*p* < 0.05 by MWU, Fig. 5b). The medical history was available for these individuals and we also found an association between vitamin B12 deficiency and ΔSD (*p* < 0.05 by MWU, Fig. 5c). There were no other associations that reached significance.

## Discussion

We have shown that we can measure B-cell diversity from peripheral blood samples by a relatively simple method of PCR and analysis of the resulting spectratype. Some very old individuals show a dramatic reduction in B-cell diversity and this is linked to frailty.

The link between the general health status of the individuals in this study and their B-cell diversity is quite striking. Since the general classification of ‘healthy’ was that the volunteers were not medicated, institutionalized or demented (Wikby*et al.*, 2002), the definition of ‘frail’ in this instance goes well beyond just immune frailty and susceptibility to infections. Thus, a collapse in B-cell diversity seems to be a marker of overall frailty rather than being specifically related to immune frailty. The complexity of old age health makes it difficult to determine whether the loss of B-cell diversity is a cause of frailty, or is a consequence of a decline in health from other causes. With subjects of such old age the medical histories could be quite complex, with co-morbidity being a common occurrence. However, with the exception of vitamin B12 deficiency, there were no significant associations between ΔSD and any particular illness. There was a significant difference in ΔSD between those patients who were deficient in vitamin B12 and the others (*p* = 0.026 by MWU). This may be of relevance since vitamin B12 deficiency can result in leucopenia (Andrès *et al.*, 2006). Since many old people are vitamin B12 deficient, and supplementation is a relatively simple treatment, it is important to elucidate the relevance of this finding. Further studies would be needed to determine whether there is a causative link between vitamin B12 deficiency and loss of B-cell diversity, and whether leucocyte diversity can be improved by restoration of normal vitamin B12 levels.

A reduced B-cell diversity is hypothesized to occur if the bone marrow output of new cells is reduced. Since the overall number of B cells does not change, reduced input of new B cells would result in a greater proportion of antigen-experienced cells in the population. The nature of a B-cell response, where the B-cell population with receptor specific for antigen expands, means that a repertoire made up of more antigen-experienced cells would be expected to show evidence of clonality. This would be evident in spectratypes, since expansion of one particular B-cell clone means expansion of a particular size of CDR3 region. To confirm this hypothesis, we sequenced the CDR3 regions from a selection of samples and showed that we did indeed see evidence of clonally related cells in cases where the spectratype was distorted, but not in cases where the spectratype was normally distributed. It is not known whether bone marrow output of B cells is reduced in old age in humans, but it has been shown for mice (Cancro, 2005). It has also been shown that, both in mice and humans, the proportion of memory B cells increases with age (Williams *et al.*, 2000; Macallan *et al.*, 2005; Colonna-Romano *et al.*, 2006).

An alternative explanation for seeing distorted spectratypes of B cells is of pathological expansions of B cells, such as are seen in leukaemia/lymphoma/monoclonal gammopathy. Individuals with such a diagnosis were not included in this study; however, the PCR method was originally developed as a sensitive test for leukaemia or lymphoma and therefore we may pick up pre-clinical conditions. An increase in monoclonal expansions of B cells, both of CD5^+^ and CD5^−^ phenotype, has previously been reported in older people (Ghia *et al.*, 2004). Monoclonal gammopathy of undetermined significance (MGUS) is a predominant plasma-cell disorder, is characterized by an increase in presence of serum monoclonal Ig, and has been shown to increase with age (Ligthart *et al.*, 1990; Kyle *et al.*, 2006). Since our cohort is very old, it is possible that MGUS accounts for some of the observed repertoire restriction with increasing age. However, our data do not suggest a high prevalence of such monoclonal expansions, the restricted repertoires that we see have a more oligoclonal appearance.

There was some correlation between our measures of B-cell diversity and the IRP status of the subjects; of the seven subjects classified as IRP positive, six had a ΔSD greater than 1. However, the ΔSD appears to be a stronger predictor of health status than IRP in this study. Six out of seven IRP-positive subjects were classified as frail as compared with 13 out of 14 subjects with a ΔSD greater than 1 being classified as frail (confirmed by ROC curve analysis, data not shown).

One immediate question arising from these findings is whether a reduced B-cell diversity affects the ability of the immune system to respond to challenge. We are commencing a study to determine whether the quality of response to vaccination is related to B-cell diversity. Since this method of analysis is relatively simple, we could envisage its future use as a predictor of the quality of response to vaccines in older people, thus enabling the identification of poor responders so that alternative healthcare strategies can be considered.

## Experimental procedures

Blood samples from cadaver donors, whose relatives had given permission for general research use, were collected as part of the normal tissue typing laboratory sampling procedures at Guy's Hospital, London, UK. Blood samples from old volunteers were previously collected as part of the Swedish NONA–Immune longitudinal study (Wikby *et al.*, 2002). Consent was obtained according to the guidelines of the ethics committee of Linköping University, Sweden.

DNA was extracted from peripheral blood mononuclear cells using the QIAamp DNA blood mini kit (Qiagen, Crawley, UK) and used at a concentration of approximately 100 ng µL^−1^ in PCRs. The CDR3 region of the rearranged IGH gene (Fig. 1a) was amplified by adding 2 µL of sample in a total volume of 25 µL using two rounds of PCR in a seminested fashion (Diss *et al.*, 1993) with the following primers: Fw3 region 5′ primer, 5′-ACACGGCTGTGTATTACTGT-3′, IGHJ region 3′ primer 1, 5′-ACCTGAGGAGACGGTGACCAGGGT-3’, IGHJ region 3′ primer 2, 5′-GTGACCAGGGTTCCTTGGCCCCAG-3′. The 3′ nested J region primer 2 was labelled with the fluorescent marker 6-Fam. PCR products were prepared for loading by adding 2 µL of blue loading dye, containing formamide, and 1 µL of 1 : 2 diluted size standard (350-TAMRA; Applied Biosystems, Warrington, UK) to 1 µL of PCR product. Tubes were heated to 95–100 °C for between 2 and 5 min and then placed onto ice to denature the DNA. The samples were run on a 6% denaturing polyacrylamide slab sequencing gel on the ABI 377, according to manufacturer's instructions (Applied Biosystems). The results were saved to file and analysed using GeneScan (Applied Biosystems) to determine the peak sizes for each main peak in the spectratype. Since the sequencing gel resolves to 1 bp, but the main peaks are every 3 bp (reflecting the predominance of open reading frame rearrangements of IGH), we took the values for the peaks at 3-bp intervals for further analysis. These values were copied into Microsoft Excel files which were imported and analysed further in the R statistical programming environment (http://www.r-project.org, R Development Core Team, 2006) to determine the standard deviation of the spectratypes, calculate MWU values, and perform ROC curve analysis. We have illustrated our findings here using the standard deviation as the metric to summarize the distributional distortions because it makes no assumptions regarding the underlying distribution. However, broadly similar conclusions were obtained when using skew, kurtosis or a direct measure of correlation to normality as the summarization metric (data not shown).

CDR3 PCR products from some of the spectratypes were sequenced by LGC Agowa sequencing services after first subcloning into the p-GEM®-T easy plasmid vector according to manufacturer's instructions (Promega, Southampton, UK). Sequences were analysed to identify IGHD and IGHJ usage and aligned using IMGT V-quest (Brochet *et al.*, 2008) and MegAlign software (from the DNAStar software suite, Lasergene, Madison, Wisconsin, USA).
